# Implementation of a visual raise hand function in PowerPoint^®^

**DOI:** 10.3205/zma001406

**Published:** 2021-01-28

**Authors:** Ivo Volf

**Affiliations:** 1 Medizinische Universität Wien, Institut für Physiologie, Vienna, Austria

**Keywords:** raise hand function, PowerPoint, remote teaching

## Abstract

**Objective: **Replacing face-to-face lessons by remote teaching due to COVID-19 led to a markedly reduced interaction between students and lecturers. In our opinion, one of the main reasons for this is the raise hand function of the respective web conference systems, which (independent of the system used) results in an unobtrusive signal that can easily be missed by the lecturer. Given the necessary focus on one's own presentation, questions can therefore only be perceived with a considerable time delay and can only be integrated into the lessons to a limited extent.

Thus, the idea arose to display question requests of the auditorium by a clear visual signal in PowerPoint® itself.

**Methodology: **With Visual Basic for Applications (VBA), Microsoft PowerPoint^®^ holds an integrated programming language that extends its functionality. Accordingly, VBA was used to program a routine running in the background of the presentation, which periodically retrieves the contents of a web-based “signal file” in a cycle of a few seconds. The content of this signal file, in turn, can be modified by the students by calling up an URL (i.e. from any Internet-capable device) - this results in a (customizable) visual signal in PowerPoint® that is temporarily visible and does not further interfere with the presentation.

**Conclusion: **With the concept presented here, a raise hand function was realized in PowerPoint®, which manifests itself as a clear visual signal independent of the web conferencing system used. This enables the lecturers to respond instantly to questions from the audience during live transmission of lectures.

## Zusammenfassung

**Zielsetzung: **Die durch COVID-19-bedingte Abhaltung von Vorlesungen als Live-Übertragungen führte zu einer im Vergleich zur Präsenzlehre deutlich reduzierten Interaktion der Studierenden mit den Vortragenden. Ein wesentlicher Grund dafür lag aus unserer Sicht in der Handhebe-Funktion der jeweiligen Webkonferenzsysteme, welche (unabhängig vom verwendeten System) in einem für den Vortragenden weitgehend unauffälligen Signal resultiert. Bei der notwendigen Konzentration auf die eigene Präsentation können Fragenwünsche somit nur mit beträchtlicher zeitlicher Verzögerung wahrgenommen und nur eingeschränkt in den Unterricht integriert werden.

Somit entstand die Idee, Fragewünsche des Auditoriums durch ein deutliches visuelles Signal in PowerPoint^®^ selbst anzuzeigen.

**Methodik: **PowerPoint^®^ verfügt mit Visual Basic für Applikationen (VBA) über eine Programmiersprache, mit welcher sich seine Funktionalität stark erweitern lässt. Entsprechend wurde VBA verwendet, um eine im Hintergrund der Präsentation ablaufende Routine zu programmieren, welche im Zyklus weniger Sekunden den Inhalt einer webbasierten „Signaldatei“ abruft. Der Inhalt dieser Signaldatei wiederum kann durch Aufruf eines URLs (also von jedem internetfähigen Gerät) von den Studierenden modifiziert werden – daraus resultiert ein (individuell gestaltbares) visuelles Signal in PowerPoint^®^, welches temporär sichtbar ist und den Ablauf der Präsentation nicht beeinträchtigt.

**Schlussfolgerung: **Mit dem hier vorgestellten Konzept wurde eine Handhebe-Funktion in PowerPoint realisiert, welche sich unabhängig vom verwendeten Webkonferenzsystem als klares visuelles Signal manifestiert. Dies ermöglicht den Lehrenden auch bei der Live-Übertragung von Vorlesungen die spontane Interaktion mit Fragewünschen des Auditoriums.

## 1. Introduction

Due to the COVID-19 related restrictions to teaching in presence, it was necessary to implement alternative teaching formats within short time.

At the Medical University of Vienna – there were no guidelines or recommendations concerning the choice between live-transmission and video-recording of lectures – the format of live-transmissions was mainly used by those lecturers who were aware of the didactic benefit of even basic interaction between students and lecturer. 

As technical platforms for the transmission of the teaching content, several web conference systems (that had already been available but were not used for teaching purposes) were used until Webex (Cisco) was licensed.

The basic concept for live transmission of lectures was independent of the web conference system used. Since the basic principle of operation is sharing one's own screen (and thus the respective presentation), the integration of survey systems (audience response systems) to promote active learning could be maintained even in remote teaching.

However, the basic step of “interaction” between student and lecturer proved to be problematic: the “announcement” of requests to speak and ask/discuss concepts and contents of the lecture was (for good reason) not realized by verbal interruption of the lecturer. Instead, the notification possibilities provided in the respective conference systems (raise hand feature) were used. These, in turn, generally provide a signal to the lecturer that is largely inconspicuous. Thus, it is perceived by the lecturer at best with a considerable delay – given that the lecturer is focused on the presentation and must assume the roles of lecturer and moderator in one person – tasks that, e.g. at scientific conferences, are fulfilled by at least two different persons. Questions transmitted via the chat function are usually presented in a more conspicuous way, but can only be read and answered at the end of the lecture or during breaks. This limits the didactic concept to encourage (and react to) spontaneous questions.

Therefore, the idea was to display question requests by the audience with a clear visual signal without influencing the flow of the presentation. 

## 2. Project description

Since live transmission of presentation contents is realized by sharing the (usually: PowerPoint^®^) presentation running on the PC of the lecturer, the idea was born to extend PowerPoint^®^ with the functionality to display information from the auditorium with a short but clear visual signal to the presenter (e.g. color change of a presentation element). 

The basic possibility of extending the functionality of individual Microsoft Office^®^ applications by means of self-written program code was greatly expanded from the mid-1990s onwards by the implementation of the interpreted programming language Visual Basic for Applications (VBA) which was also largely standardized between the individual applications (Word, Excel, PowerPoint, Access).

VBA in principle enables PowerPoint to access the contents of web-based files (in read and write access). Conversely, the content of such a web-based file can be manipulated by calling up a web page (pressing a button) and thus via any internet-capable device (smartphone, tablet, notebook).

Thus, such a “signal file” – placed in an accessible web directory – can serve as an interface between the auditorium and PowerPoint®, provided that students have the opportunity to manipulate the content of the signal file by calling up an appropriate URL. At the same time, PowerPoint^®^ must periodically (at intervals of a few seconds) poll the content of this file during the presentation and – in case of a modified value – respond with a visual signal (and subsequently reset the content of the signal file in order to be able to display further questions).

Such a communication interface was realized via VBA and php. Difficulties arose somewhat surprisingly from the fact that VBA in PowerPoint^®^ (in contrast to Word, Excel and Access) does not include a timer function and the periodic polling of the signal file therefore had to be solved by means of the Windows API.

## 3. Results

With the concept presented here, a raise hand function was implemented in PowerPoint^®^, whereby questions from the auditorium are clearly displayed to the lecturer with minimal time latency.

Due to the implementation in PowerPoint^®^, this can be archieved independent of the web conference system used. The presentation of the visual signal to the lecturer is freely configurable and is done via a (modifiable) object (“Shape”) placed on the first slide.

First experiences with the solution presented here showed stable performance of the application. Feedback from lecturers could only be obtained as narrative feedback due to the short-term nature of implementation (evaluations in compulsory education require approval of the data protection commission). As a result, the clear signaling was explicitly stated as helpful functionality and a more spontaneous interaction was experienced. However, this does not seem to increase students' motivation to ask their own questions.

## 4. Limitations

Due to the integration of the Windows API, the solution presented here is only executable under MS-Windows^®^. A use in combination with presenter view mode is not possible – this limitation is system-dependent and therefore cannot be fixed in the approach presented here.

## 5. Availability

All files necessary to implement the functionality described here can be used freely for academic teaching: http://educativo.at/RaiseHand/.

## Competing interests

The author declares that he has no competing interests.

